# Function Annotation of Hepatic Retinoid x Receptor α Based on Genome-Wide DNA Binding and Transcriptome Profiling

**DOI:** 10.1371/journal.pone.0050013

**Published:** 2012-11-15

**Authors:** Qi Zhan, Yaping Fang, Yuqi He, Hui-Xin Liu, Jianwen Fang, Yu-Jui Yvonne Wan

**Affiliations:** 1 Department of Gastroenterology Hepatology, Guangzhou First Municipal People's Hospital, Guangzhou Medical College, Guangzhou, Guangdong Province, China; 2 Department of Medical Pathology and Laboratory Medicine, University of California Davis, Davis Health Systems, Sacramento, California, United States of America; 3 Applied Bioinformatics Laboratory, University of Kansas, Lawrence, Kansas, United States of America; Clermont Université, France

## Abstract

**Background:**

Retinoid x receptor α (RXRα) is abundantly expressed in the liver and is essential for the function of other nuclear receptors. Using chromatin immunoprecipitation sequencing and mRNA profiling data generated from wild type and RXRα-null mouse livers, the current study identifies the bona-fide hepatic RXRα targets and biological pathways. In addition, based on binding and motif analysis, the molecular mechanism by which RXRα regulates hepatic genes is elucidated in a high-throughput manner.

**Principal Findings:**

Close to 80% of hepatic expressed genes were bound by RXRα, while 16% were expressed in an RXRα-dependent manner. Motif analysis predicted direct repeat with a spacer of one nucleotide as the most prevalent RXRα binding site. Many of the 500 strongest binding motifs overlapped with the binding motif of specific protein 1. Biological functional analysis of RXRα-dependent genes revealed that hepatic RXRα deficiency mainly resulted in up-regulation of steroid and cholesterol biosynthesis-related genes and down-regulation of translation- as well as anti-apoptosis-related genes. Furthermore, RXRα bound to many genes that encode nuclear receptors and their cofactors suggesting the central role of RXRα in regulating nuclear receptor-mediated pathways.

**Conclusions:**

This study establishes the relationship between RXRα DNA binding and hepatic gene expression. RXRα binds extensively to the mouse genome. However, DNA binding does not necessarily affect the basal mRNA level. In addition to metabolism, RXRα dictates the expression of genes that regulate RNA processing, translation, and protein folding illustrating the novel roles of hepatic RXRα in post-transcriptional regulation.

## Introduction

Retinoid x receptor (RXR) plays a critical role in metabolism, development, differentiation, proliferation, and cell death by regulating gene expression [1], [2]. The expression profile of RXRs and their downstream signaling is altered in a variety of diseases including breast cancer [3] and viral hepatitis [4]. Correspondingly, RXR agonists are implicated in cancer prevention, antiviral therapy, dermatological disease, and metabolic syndromes [2], [5], [6]. Mediated via RXRs (α, β, and γ) and retinoic acid receptors (α, β, and γ), retinoic acid exerts its biological effects. In addition to RARs, RXRα is crucial for many other receptors to work. The receptors for fatty acids, oxysterols, bile acids, vitamin D, etc. form dimers with RXRs to regulate gene transcription. RXRα is essential for fetal morphogenesis [7]. Global RXRα knockout is embryonically lethal and the embryos develop myocardiac hypoplasia with reduced liver size at gestational day 12.5 [8]. RXRα is predominantly expressed in the liver [7]. Hepatocyte RXRα knockout mice were produced to study its role in the liver [9]. RXRα-deficient mice have compromised lipid [10], carbohydrate [11], xenobiotic [12], and amino acid homeostasis [13]. In addition, hepatocyte RXRα is also implicated in liver steatosis and inflammation as well as regeneration [14]–[17]. Specifically, hepatocyte RXRα-deficient mice are more susceptible than wild type mice to alcohol and non-alcohol-induced steatohepatitis [13]–[15]. Partial hepatectomy-induced liver regeneration is also hampered due to hepatic RXRα deficiency [16]. Liver is the organ that stores and converts retinol to its biological active form i.e. retinoic acid. Liver also produces binding proteins to deliver retinoic acid to target sites. Furthermore, hepatocytes express high level of RXRα. Thus, it is pivotal to understand the biological function of RXRα in the liver.

The current study determines genome-wide RXRα binding in normal mouse livers by chromatin immunoprecipitation using specific anti-RXRα antibody followed by next generation sequencing (ChIP-seq). In addition, microarray was performed to identify genes that are differentially expressed in wild type and RXRα-null mouse livers. Combining the two datasets, we established the relationship between hepatic RXRα-DNA binding and RXRα-dependent gene expression. This global profiling of RXRα binding along with gene expression not only allows us to capture all RXRα downstream targets and pathways, but also helps us to understand the molecular mechanism by which hepatic RXRα regulates gene expression.

**Table 1:** Comparison of RXRα Binding Sites.

Nuclear Receptor	Target Gene	Our data location (motif)	Reported data location (motif)	Reference
RARs	Prcka	−19 (NK)	−93∼−65 (NR)	[23]
	Cyp26a1	−1862 (DR5)	−2 kb (DR5)	[24]
	RARβ	−341 (DR5)	−59 (DR5)	[25]
FXR	Nr0b2	−271 (IR1, DR3)	−320∼−220 (NR)	[26]
	Abcb11	−220 ∼ 250 (IR1)	−240∼−140 (NR)	[26]
LXR	Abca1	−183 (DR3, 4)	−70 (DR4)	[27]
	Fasn	−658 (DR4)	−660 (DR4)	[28]
	Pltp	−2.2 (ER3)	−2.6 kb (DR4)	[29]
PPARs	ACOX1	−286 (NK)	−550 (DR1)	[30]
	ALDH3A2	−4.69 kb (DR1)	−4.63 kb (DR1)	[31]
	Nfkbia	−111 (NK)	≥−1.9 kb (DR1)	[32]
PXR	Cyp3a11	−1.5 kb (ER1)	−1.5 kb (NR)	[33]
	Slc01a4	−10 kb (DR3, ER3)	−10 kb (NR)	[33]
	Abcc3	3.8 kb (IR3, DR4)	3.8 kb (NR)	[33]
CAR	Cyp2b10	−2.3 kb (DR4, ER1)	−2.3 kb (NR)	[34]
	Abcc2	−97 (NK)	−400 (ER8)	[35]
VDR	Spp1	−701 (DR3)	−761 (DR3)	[36]
	Cyp24a1	−322 (NK)	−265 (DR3)	[37]
	Pckra	114 kb (ER3, DR1)	127 kb (NR)	[38]
TR	Cyp7a1	−3 kb (DR0)	−3 kb (DR0, DR4)	[39]
	Thrsp	−1375 (DR0, IR4)	−1385 (NR)	[40], [41]
	Egr	−90 (IR5)	−112∼−77 (IR)	[42]

DR, direct repeat; ER, everted repeat; Hu, human; IR, inverted repeat; kb, kilo-base pair; Ms, mouse; NK, not known NR, not reported.

## Materials and Methods

### Animals and Tissues

The generation of hepatocyte RXRα-deficient mice was described in previous publications [9], [10]. The *LoxP* sequences were inserted into introns flanking the fourth exon of the RXRα gene and DNA binding domain of the gene is deleted after crossing the *floxed RXRα* allele against a transgenic line in which the *cre* recombinase is expressed under the control of the albumin promoter. The mutant mice express a truncated protein that lacks the DNA binding domain. However, the ligand binding domain remains intact. Mouse livers were collected at 10 AM from 12-week-old wild type (WT) and hepatocyte RXRα knockout (KO) mice, which have C57BL/6 genetic background. Tissues were snap frozen in liquid nitrogen and kept at −80°C for future use. Animal protocols and procedures were approved by the Institutional Animal Care and Use Committee (IACUC) at the University of Kansas Medical Center and the University of California, Davis.

**Figure 1:**
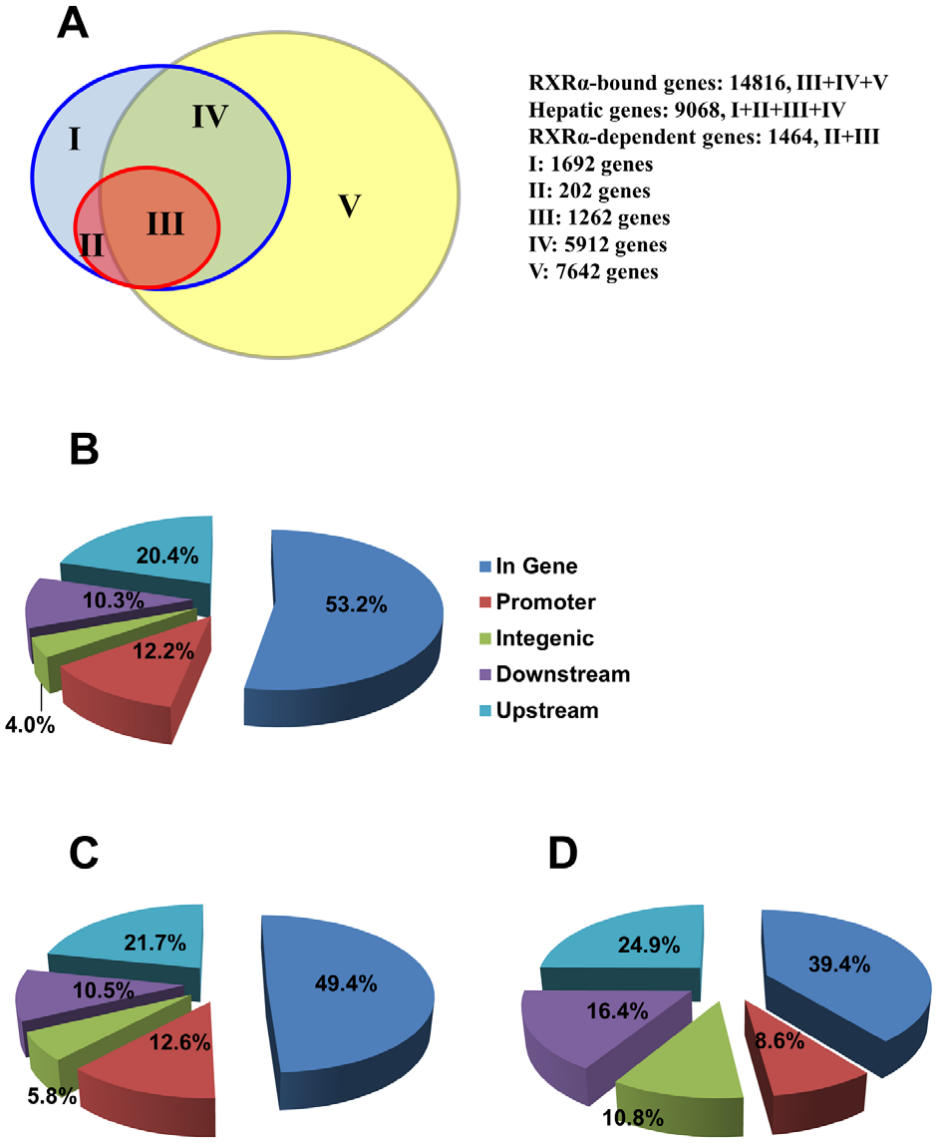
Global analysis of ChIP-seq and microarray data. (A) Venn diagrams of RXRα-bound genes (III+IV+V), hepatic genes (I+II+III+IV), and RXRα-dependent genes (II+III)**.** RXRα binding location for (B) RXRα-dependent, (C) RXRα-independent hepatic expressed, and (D) non-hepatic expressed genes. In gene: peak summit located within the coding region; Promoter: peak summit located within −2 kb ∼0 bp to the transcription start site (TSS); Upstream: peak summit located within -100 kb∼−2 kb to the TSS; Downstream: peak summit located within 0 bp ∼100 kb to 3′ end of the gene; Intergenic: peak summit located outside the above mentioned regions. Red circle: RXRα-dependent genes; Blue circle: hepatic genes; Yellow circle: RXRα-bound genes. I: RXRα-independent genes and lack of RXRα binding; II: RXRα-dependent genes but lack of RXRα binding; III: RXRα-dependent genes that have RXRα binding; IV: RXRα-independent genes that have RXRα binding; V: non-hepatic genes that have RXRα binding.

### RNA Preparation and Microarray

Total RNA was extracted using TRIzol Reagent (Invitrogen Co., CA) and purified with the RNeasy Mini Kit (Qiagen Inc., CA). The quantity and quality of the total RNA were assessed by Bioanalyzer 2100 (Agilent Technologies, CA). Complementary DNA was made using High Capacity RNA-to-cDNA Kit (Applied Biosystems, CA). Affymetrix chips (MOE 430A 2.0) that covered about 14,000 mouse genes were used. Microarray (n = 3 per group) and data processing as well as the methods used for data validation were described in our previous publication [18]. By Student *t*-test, genes differentially expressed in wild type and RXRα-knockout mouse livers with *p* value less than 0.05 were identified as RXRα-dependent genes.

**Figure 2:**
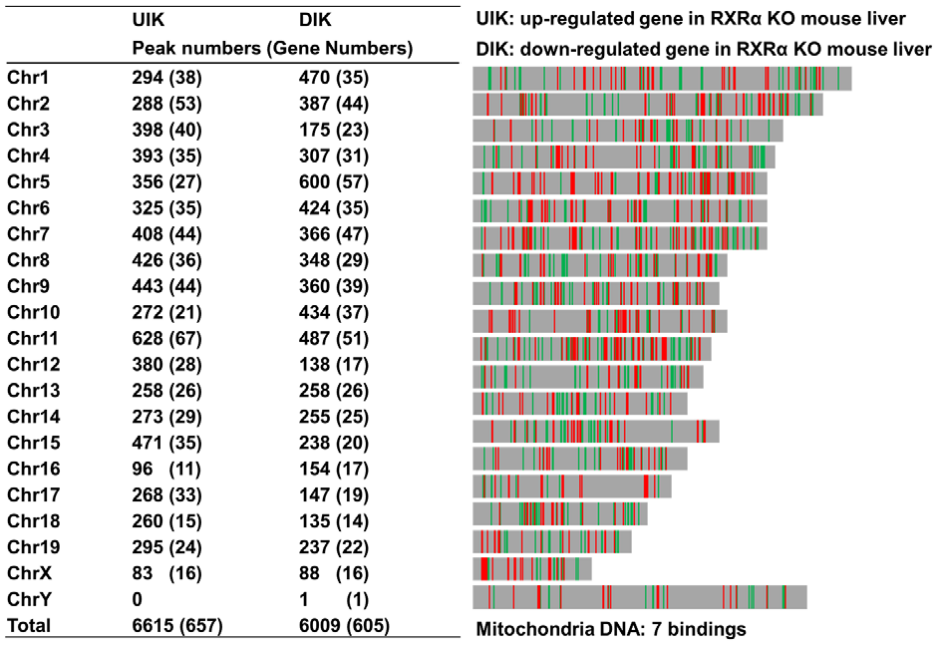
Chromosomal distribution of RXRα peaks in RXRα-dependent genes in mouse liver. Each bar represents an RXRα binding site on the mouse genome. UIK (green): up-regulated in RXRα KO liver; DIK (red): down-regulated in RXRα KO liver.

### Chromatin immunoprecipitation sequencing (ChIP-Seq) and ChIP-qPCR

Frozen livers were fixed in 1% formaldehyde (pH = 7) for 15 minutes before being quenched with 0.125 M glycine. Following cell lysis, the nuclear fraction was extracted and sonicated to produce 300–500 base-pair (bp) DNA fragments. Genomic DNA (Input) was prepared by treating aliquots of chromatin with RNase, proteinase K and heated for de-crosslinking, followed by ethanol precipitation. Chromatin (30 µg) was precleared by Dynase beads (Invitrogen Co., CA) before incubation with a ChIP-quality anti-RXRα antibody (Santa Cruz, CA). An antibody to IgG (Santa Cruz, CA) and RNA Pol II (Millipore, MA) was used as negative and positive control, respectively. Samples were incubated with prepared Dynase beads at 4°C overnight, followed by de-crosslinking and purification. DNA fragment library was size-selected (175–225 bp) on an agarose gel. Amplified DNAs (DNA library) were sequenced on the Illumina Genome Analyzer II. For ChIP-seq data validation, DNA fragments generated based on above mentioned method (n = 3) were quantified by real-time PCR with Power SYBR® Green PCR Master Mix (Life Technologies Co., CA).

**Figure 3:**
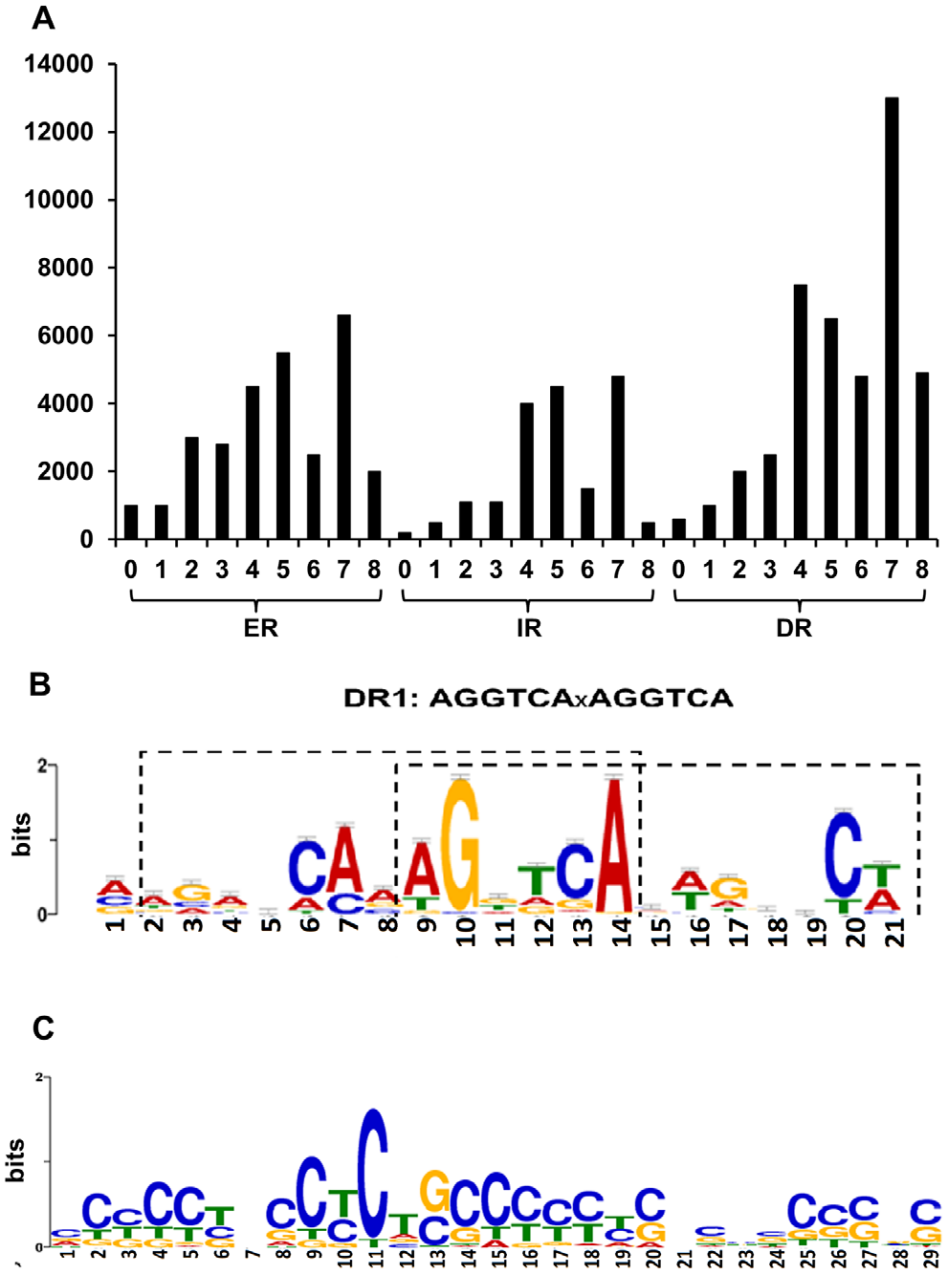
Motif Analyses. (A) Global profiling of RXRα binding motifs in mouse liver genome predicted by Hidden Markov Model. DR: direct repeat; ER: everted repeat; IR: inverted repeat. (B) Out of the top 500 strongest bindings, the most common motif contains three half nuclear receptor binding sites, which may form two overlapped DR1s sharing the middle half site. (C) The other common motif contains a GC box that matches to the Sp1 binding site.

**Table 2:** Biological Functional Pathway Analysis of RXRα-dependent Genes.

	Gene Numbers				
Biological Processes* (Gene Number)	RXRα-Bound (%)	UIK^#^	DIK^#^	*p* value	Bonferroni
oxidation reduction (120)	109 (90.8)	65	55	1.50E-18	4.40E-15
translation (50)	24 (48.0)	10	40	1.30E-05	3.70E-02
lipid biosynthetic process (48)	42 (87.5)	32	16	5.40E-07	1.60E-03
generation of precursor metabolites and energy (47)	40 (85.1)	27	20	9.70E-08	2.80E-04
fatty acid metabolic process (41)	39 (95.1)	19	22	1.50E-09	4.40E-06
steroid metabolic process (36)	31 (86.1)	26	10	1.60E-08	4.70E-05
cofactor metabolic process (36)	33 (91.7)	20	16	3.90E-07	1.10E-03
carboxylic acid biosynthetic process (29)	28 (96.6)	18	11	2.90E-06	8.50E-03
organic acid biosynthetic process (29)	28 (96.6)	18	11	2.90E-06	8.50E-03
coenzyme metabolic process (29)	29 (100)	17	12	3.90E-06	1.10E-02
protein folding (26)	15 (57.7)	10	16	1.20E-05	3.30E-02
electron transport chain (24)	21 (87.5)	14	10	1.20E-05	3.50E-02
steroid biosynthetic process (22)	17 (77.3)	17	5	4.80E-08	1.40E-04
sterol metabolic process (22)	18 (81.8)	18	4	2.20E-07	6.50E-04
anti-apoptosis (20)	18 (90.0)	6	14	3.30E-05	9.30E-02
cholesterol metabolic process (19)	15 (78.9)	15	4	4.10E-06	1.20E-02
sterol biosynthetic process (14)	10 (71.4)	14	0	1.10E-07	3.30E-04
cholesterol biosynthetic process (11)	7 (63.6)	11	0	3.30E-06	9.50E-03

* Biological processes were obtained from DAVID functional annotation.

# UIK: up-regulated gene in RXRα KO mouse liver; DIK: down-regulated gene in RXRα KO mouse liver.

### Data Analysis

Primary image analysis and base calling were performed using Genome Analyzer Pipeline Software (Illumina Inc., CA). All sequenced reads were aligned to mm9 mouse reference genome using bowtie version 0.12.7 [19]. Only uniquely mapped reads were included. Regions with reads enrichment were detected using the Model-based Analysis of ChIP-Seq (MACS v 1.4.1) method [20]. Non-specific peaks with false discovery ratio (FDR) greater than 0.1 were eliminated by comparing them with the IgG background. Peaks were further split by Mali Salmon's Peak Splitter (http://www.ebi.ac.uk/bertone/software.html) and filtered by *p-*value of Poisson distribution lower than 10^−5^. The peak annotation was based on the UCSC genome NCBI/mm9 database. Relative to the transcription start site (TSS) or 3′ end of the gene, the peak locations were defined as ingene (summit located within the coding region), promoter (−2 kb∼0 bp to the TSS), downstream (0∼100 kb to the 3′ end), upstream (−100 kb ∼ −2 kb to the TSS), or intergenic (more than 100 kb away from TSS or 3′ end).

**Figure 4:**
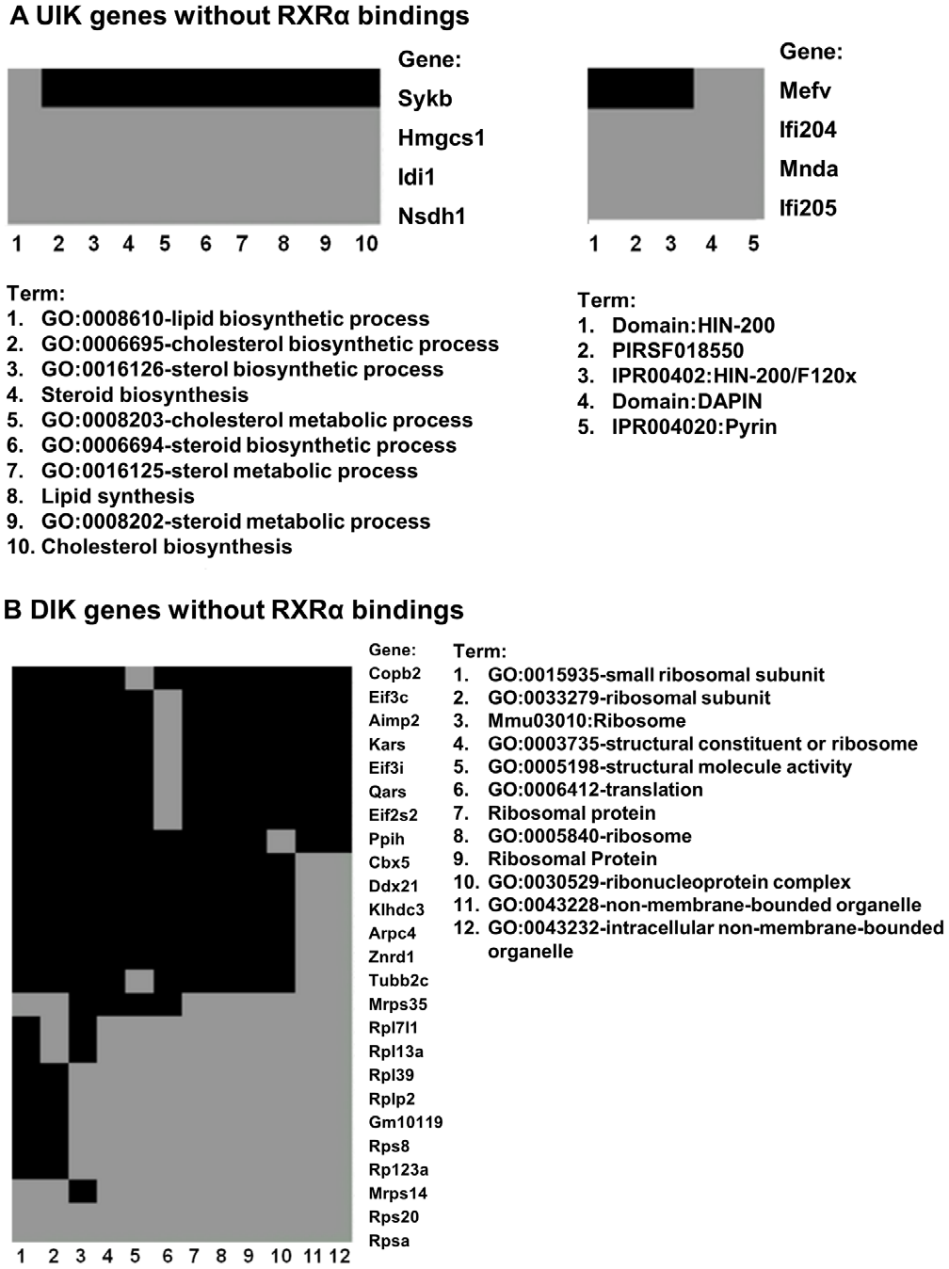
Representative heat maps of functional annotation clustering of RXRα-dependent genes without RXRα binding sites. Genes up-regulated (A) or down-regulated (B) due to hepatic RXRα deficiency were subjected to DAVID functional annotation. The gene-term association relationship was generated using the functional annotation clustering tool in the DAVID website. Gray areas indicate the gene-term associations have been established by the literatures. Black areas show the gene-term relationships can exist, but requires experimental validation. Explanation for some of the listed terms shown in A: Domain: HIN-200 is a domain of HIN-200 protein, PIRSF018550 is a protein of PIR super family with serial number of 018550, IPR004021 is a domain of protein HIN-200/IF120x. Domain: DAPIN is a domain for apoptosis and interferon response. IPR004020: Pyrin is a subclass of DAPIN domain that interacts with proteins that have pyrin domain.

**Table 3:** Biological Functional Analysis of RXRα-bound Genes.

David Biological Functional Annotation	Number of gene (%)	*p* value	Bonferroni
regulation of transcription	1605 (11.8)	3.20E-14	1.90E-10
Transcription	1329 (9.8)	7.70E-22	4.70E-18
intracellular signaling cascade	729 (5.4)	2.80E-23	1.70E-19
phosphate metabolic process	696 (5.1)	3.90E-24	2.30E-20
phosphorus metabolic process	696 (5.1)	3.90E-24	2.30E-20
protein localization	613 (4.5)	6.40E-24	3.90E-20
phosphorylation	575 (4.2)	2.20E-19	1.30E-15
oxidation reduction	559 (4.1)	3.00E-26	1.80E-22
establishment of protein localization	531 (3.9)	8.90E-20	5.40E-16
protein transport	527 (3.9)	1.20E-19	7.20E-16
protein amino acid phosphorylation	514 (3.8)	7.10E-18	4.30E-14
macromolecule catabolic process	504 (3.7)	1.80E-11	1.10E-07
positive regulation of macromolecule metabolic process	497 (3.7)	1.00E-13	6.10E-10
regulation of transcription from RNA polymerase II promoter	478 (3.5)	9.70E-12	5.80E-08
positive regulation of biosynthetic process	439 (3.2)	1.10E-12	6.60E-09
positive regulation of cellular biosynthetic process	436 (3.2)	7.20E-13	4.30E-09
protein catabolic process	436 (3.2)	5.40E-12	3.30E-08
positive regulation of macromolecule biosynthetic process	423 (3.1)	8.30E-14	5.00E-10
positive regulation of nitrogen compound metabolic process	418 (3.1)	4.10E-13	2.50E-09
positive regulation of nucleobase, nucleoside, nucleotide and nucleic acid metabolic process	407 (3.0)	2.70E-13	1.60E-09
negative regulation of macromolecule metabolic process	401 (2.9)	2.70E-12	1.60E-08
cell death	398 (2.9)	3.80E-11	2.30E-07
positive regulation of gene expression	390 (2.9)	5.90E-13	3.60E-09
positive regulation of transcription	384 (2.8)	3.70E-14	2.20E-10
intracellular transport	354 (2.6)	5.00E-15	3.00E-11
cellular response to stress	325 (2.4)	1.30E-11	7.90E-08
nitrogen compound biosynthetic process	253 (1.9)	7.40E-13	4.50E-09
cellular macromolecule localization	250 (1.8)	6.90E-12	4.20E-08
cellular protein localization	248 (1.8)	1.10E-11	6.80E-08
cofactor metabolic process	161 (1.2)	1.90E-12	1.10E-08

**Table 4:** Biological Functional Analysis of RXRα-bound non-Hepatic Genes.

David Biological Functional Annotation	Number of gene (%)	*p* value	Bonferroni
regulation of transcription	703 (10.8)	2.10E-07	9.00E-04
transcription	554 (8.5)	2.00E-05	0.084
regulation of RNA metabolic process	494 (7.6)	2.80E-08	1.20E-04
regulation of transcription, DNA-dependent	493 (7.6)	3.60E-09	1.60E-05
ion transport	253 (3.9)	3.50E-07	0.002
cell adhesion	198 (3.0)	1.20E-05	0.052
biological adhesion	198 (3.0)	1.40E-05	0.059
metal ion transport	166 (2.5)	1.10E-06	0.005
neuron differentiation	163 (2.5)	2.10E-09	9.00E-06
cell motion	138 (2.1)	9.00E-06	0.039
cell-cell signaling	120 (1.8)	1.40E-07	6.30E-04
neuron development	117 (1.8)	1.50E-06	0.006
regulation of small GTPase mediated signal transduction	101 (1.5)	3.00E-08	1.30E-04
cell part morphogenesis	87 (1.3)	1.30E-05	0.054
cell projection morphogenesis	86 (1.3)	2.60E-06	0.011
regulation of Ras protein signal transduction	77 (1.2)	9.30E-06	0.04
cell morphogenesis involved in neuron differentiation	77 (1.2)	1.20E-05	0.051
synaptic transmission	76 (1.2)	9.20E-06	0.04
neuron projection morphogenesis	74 (1.1)	2.30E-05	0.096
axonogenesis	70 (1.1)	1.70E-05	0.072
axon guidance	50 (0.8)	1.10E-06	0.005
spinal cord development	27 (0.4)	2.40E-05	0.099

### Motif and Pathway Analysis

The sequences that were 100 bp up and downstream from the summits of the top 500 peaks, which had the highest peak scores, were subjected to motif analysis using by MEME-ChIP (Multiple EM for Motif Elicitation) [21]. Furthermore, a Hidden Markov Model (HMM) was established based on nuclear receptor binding sites from published work [22] and JASPAR CORE (http://jaspar.cgb.ki.se/cgi-bin/jaspar db.pl) to predict the specific motif information for all of the RXRα binding sites. All of the biological function and pathway analyses were performed using the Functional Annotation Tool in DAVID (DAVID; http://www.david.niaid.nih.gov). Functional pathways or process with *p*<0.05 and Bonferroni value <0.1 were accepted.

**Figure 5:**
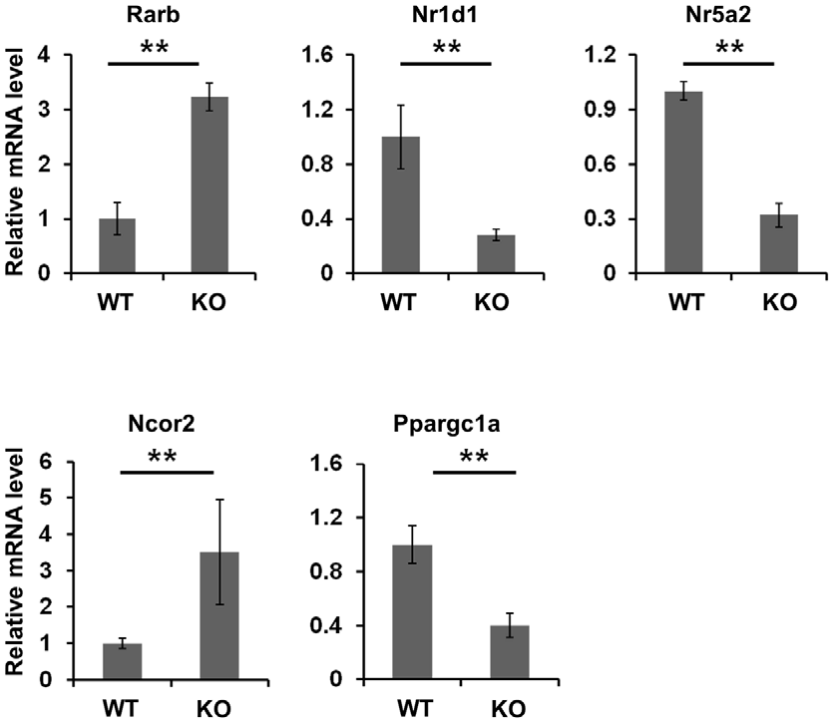
Expression of genes in wild type and RXRα-null livers. RNA extracted from wild type and RXRα-null livers (n = 3–4) were subjected to real time PCR to determine the expression level of the studied genes. Data were normalized to glyceraldehyde 3-phosphate dehydrogenase (GAPDH) mRNA level.**: *p*<0.05 in comparisons between two groups. Microarray experiment showed that fold change in Rarβ, Nr1d1, Nr5a2, Ncor2, and Ppargc1a mRNA levels due to hepatic RXRα deficiency was 1.8, 0.7, 0.5, 1.3, and 0.3, respectively (n = 3, *p*<0.05).

**Table 5:** Nuclear Receptor and Cofactor Genes that Bound by RXRα.

Endocrine receptors	Orphan nuclear receptors			Nuclear
				Receptor cofactors
Ar	Hnf1a	Rara	Nr1d1 (Rev-erbα)*	Ppargc1a*
Nr3c1 (Gr)	Hnf1b	Rarb*	Nr1d2(Rev-erbβ)	Ppargc1b
Nr3c2 (Mr)	Hnf4a	Rarg	Nr2c1 (Tr2)	Ncor2*
Esr1	Nr1h3 (Lxr)	Rxra	Nr2c2 (Tr4)	Thrap3
Esr2	Nr1h4 (Fxrα)	Rxrb	Nr2e3 (Pnr)	
Pgr	Nr1h5 (Fxrβ)	Rxrg	Nr2f1 (COUP-TF1)	
	Nr1i2 (Pxr)	Thra	Nr2f2 (COUP-TF2)	
	Nr1i3 (Car)	Thrb	Nr2f6 (COUP-TF3)	
	Ppara	Rora	Nr4a1 (Nur77)	
	Ppard	Rorc	Nr4a3 (Nor1)	
	Pparg	Ror1	Nr6a1 (Gcnf)	
	Esrra	Ror2	Nr0b2 (Shp1)	
	Esrrb	Nr5a1		
	Esrrg	Nr5a2*		

* : RXRα-dependent genes confirmed by real-time PCR.

### Statistical Analysis

For ChIP-qPCR data and microarray data, the difference between two groups was analyzed by Student's *t* test. *P*<0.05 was considered statistically significant.

## Results

### Validation of RXRα binding sites discovered by ChIP-seq

Genes with known RXRα heterodimer response elements were found to be bound by RXRα at similar locations in our ChIP-seq data to what has been previously reported (Table 1). To further validate the ChIP-seq results, RXRα binding sites with various peak scores were randomly selected for confirmation by ChIP-qPCR. Peak scores were used to scale the strength of RXRα binding. The results showed 100%, 88%, and 87.5% confirmation for peaks that had strong (peak score ≥200), medium (100≤ peak score <200), and weak (peak score <100) bindings, respectively [23]–[42].

### Global RXRα binding and RXRα-dependent gene expression in mouse livers

Microarray data showed that there were 9068 genes with detectable signals (mRNA levels), which are referred to as “hepatic genes”. Among these, 768 were significantly up-regulated (UIK: up-regulated in KO) and 696 genes were down-regulated (DIK: down-regulated in KO) in the hepatocyte RXRα KO mouse livers. Thus, 16.14% of hepatic genes (1464 out of 9068) were expressed in an RXRα-dependent manner.

There were 109971 confident RXRα peaks detected in 14816 genes in the mouse liver. The average is 7.4 peaks per gene. Remarkably, 79.1% (7174) of the hepatic genes were bound by RXRα (Figure 1A), which includes 657 (85.5%) UIK and 605 (86.9%) DIK genes. However, 7642 RXRα-bound genes did not have significant signals by microarray in either WT or RXRα KO mouse livers and we refer to those genes as “non-hepatic genes”.

The frequencies of promoter peaks are similar for RXRα-dependent (12.2%) and -independent (12.6%) genes (Figure 1B and C). RXRα binding sites occurred more frequently in promoter (12.6% *vs.* 8.6%) and coding (49.4% *vs.* 39.4%) regions for the hepatic than non-hepatic genes (Figure 1C and D). Conversely, RXRα bound more frequently to the intergenic, up- and downstream regions for the non-hepatic than the hepatic genes. Thus, it seems that the binding location of RXRα could be a determinant factor for the level of hepatic gene expression.

In terms of chromosomal distribution, RXRα binding occurred more frequently on chromosome 5 and 11 (956 and 1115 RXRα binding sites, respectively) than others (Figure 2). Notably, gene function analysis demonstrated that 32% of RXRα-bound genes on chromosome 5 are associated with alternative splicing, while 52% of RXRα-bound genes on chromosome 11 encode phosphor-proteins suggesting the role of RXRα in post-transcriptional modification. In addition, seven RXRα binding sites (peak scores ranging170–470) were mapped on mitochondrial DNA in the areas where genes were not identified by the UCSD Gene Browser. 171 peaks were matched on X chromosome, while only 1 peak was noted on Y chromosome.

### Motif Analysis of RXRα binding sites

Since RXRα dimerizes with multiple nuclear receptors, its binding sites are composed of diverse binding motifs. The result predicted by the Hidden Markov Model identified DR1 (12.5%) as the most common motif for RXRα binding in mouse liver followed by DR4, inverted repeat (IR) 1, DR3, and IR3. Furthermore, spacers 1, 3, and 4 were relatively more prevalent than other spacers (Figure 3A). The results of the MEME-ChIP analysis for the 500 strongest bindings demonstrated that one of the most common motifs contains three half nuclear receptor binding sites, which may form two overlapped DR1s (*p* = 7.52E-6) (Figure 3B). The other identified sequence contains a GC box that matches to the motif of specific protein 1 (Sp1) binding site (*p* = 2.28E-5) (Figure 3C) [43].

### Functional analysis of RXRα based on genetic profiling of hepatocyte RXRα-deficient mouse livers

Biological functional analysis of both UIK and DIK genes demonstrated that RXRα-dependent genes participated predominantly in oxidation/reduction, lipid metabolism, generation of precursor metabolites and energy, cofactor metabolism, carboxylic acid biosynthesis, organic acid biosynthesis, coenzyme metabolism, protein folding, electron transport chain, translation and apoptosis. Among these, an average of 84% of the genes was bound by RXRα (Table 2). The ratio of UIK and DIK genes in each pathway revealed a distinct role for hepatic RXRα in regulating lipid metabolism and apoptosis. For example, all of the sterol and cholesterol biosynthetic-related genes and most of the steroid and lipid biosynthetic-related genes were up-regulated in KO mouse livers, which indicate the role of hepatic RXRα in the metabolism of sterol and cholesterol. In the apoptosis pathway, 14 out of 20 anti-apoptotic genes were significantly down-regulated in KO livers, whereas only 6 were up-regulated. This finding suggests the pro-apoptotic role of hepatocyte RXRα. In addition to serving as a transcriptional factor, RXRα has a role in regulating translation as many translation-related genes were down-regulated due to RXRα deficiency.

There were four UIK genes, including spleen tyrosine kinase (Sykb), 3-hydroxy-3-methylglutaryl-Coenzyme A synthase 1 (Hmgcs1), isopentenyl-diphosphate delta isomerase (Idi1), and NADP dependent steroid dehydrogenase-like (Nsdhl), which were not bound by RXRα and are also involved in steroid and cholesterol metabolism, suggesting RXRα could directly and indirectly regulate steroid and cholesterol homeostasis (Figure 4A). The analysis also revealed that the pyrin-related genes including Mediterranean fever (Medv), myeloid cell nuclear differentiation antigen (Mnda), and interferon activated genes 204 and 205 (Ifi204, Ifi205) were significantly induced due to lack of hepatic RXRα, but they do not have RXRα binding sites (Figure 4A). In addition, there were 25 DIK genes without RXRα binding sites, which were functionally related to intracellular non-membrane-bounded organelle, ribonucleoprotein complex, ribosome, translation or structural molecule activity (Figure 4B). Additionally, biological functions of RXRα-bound genes (Table 3) and RXRα-bound non-hepatic genes (Table 4) were analyzed. The RXRα-bound genes were implicated extensively in metabolic process, gene expression, protein processing, cell death, and response to stress, which encompassed most of the functional pathways of RXRα-dependent genes. However, the analysis of RXRα-bound non-hepatic genes revealed the potential role of RXRα in other tissues, such as neuron differentiation and development, cell adhesion, motion, and morphogenesis as well as signal transduction.

### The interaction between RXRα and other nuclear receptors as well as cofactor genes

Surprisingly, many steroid and orphan nuclear receptor genes were bound by RXRα in mouse livers (Table 5). Among these, the mRNA levels of the retinoid acid receptor beta (Rarb), nuclear receptor 1d1 (Nr1d1, Rev-erbα), and Nr5a2 was significantly changed due to hepatic RXRα deficiency. These findings were validated by real-time PCR in livers (Figure 5). Notably, some nuclear receptor cofactors, such as peroxisome proliferative activated receptor γ coactivator 1 α (Ppargc1a), β (Ppargc1b), and nuclear receptor co-repressor 2 (Ncor2), were also bound by RXRα and Ppargc1a and Ncor2 was also expressed in an RXRα-dependent manner (Figure 5).

## Discussion

It has been more than two decades since the cloning of RXRα. Accumulated literature clearly indicates the importance of this nuclear receptor in regulating liver disease processes such as metabolic syndrome, alcoholic liver disease, chronic hepatitis C, and liver cancer, which comprise some of the most serious worldwide health issues today [2], [4], [5]. Thus, a global profiling of the bona fide RXRα genomic binding sites has become essential in order to illustrate the function and underlying regulatory mechanism mediated by RXRα. Using high throughput genomic methods and the knockout mouse model, the current study not only identifies the *in vivo* interaction of RXRα and the mouse genome, but also establishes the relationship between binding and hepatic gene expression as well as biological pathways. The hepatocyte RXRα-deficient mice were generated by deleting the DNA binding domain of the RXRα [9]. Thus, the differential gene expression between WT and KO mouse livers is due to lack of direct DNA binding of RXRα. Our data indicated that RXRα bound 78.7% of hepatic expressed genes and more than 80% of RXRα-dependent genes revealing the direct extensive role of RXRα in regulating liver gene expression and liver function. Additionally, 202 RXRα-binding free genes also displayed an RXRα-dependent expression pattern. These genes could be regulated by RXRα indirectly or post-transcriptionally. Moreover, RXRα binding sites were located in all of the mouse chromosomes as well as the mitochondrial DNA. However, the enriched bindings on chromosome 5 and 11 and rare bindings on Y chromosome suggest specific biological functions of RXRα. The current study also presented evidence for the potential role of RXRα in regulating mitochondrial gene function. Since mitochondria DNA is maternally derived, the finding is congruent with the specific binding of RXRα to the X chromosome. These observations suggest a gender-specific effect of RXRα. Taken together; the data indicate the extensive biological action of endogenous RXRα ligands such as retinoic acid and fatty acids in the liver. RXR has three isoforms. Among them, RXRα has the highest expression level in the liver. It would be important to study the differential and redundant role of RXRβ and γ using the same approach.

Promoter and enhancer regions are regarded as the most important regulatory regions in the control of transcription [44]. Comparing the distribution of RXRα binding sites in non-hepatic, hepatic, and RXRα-dependent genes, RXRα bindings occurred more frequently within the coding and promoter regions for hepatic than non-hepatic genes. However, the distribution profile of RXRα binding was similar between RXRα-dependent and -independent genes. Thus, it seems like direct binding of RXRα does not necessarily affect baseline mRNA levels. There are several potential reasons for this. Firstly, the effect of RXRs might be redundant and the presence of RXRβ and γ in hepatocyte RXRα-deficient mouse liver may be sufficient to maintain the basal transcriptional machinery of the hepatic RXR target genes. Secondly, albumin-*cre* recombinase was used to produce the RXRα knockout mice. Since albumin is predominantly expressed in the hepatocyte, it is also possible that some of those RXRα-bound genes are predominantly expressed in other types of liver cells rather than hepatocytes. Thus, RXRα deficiency does not alter the hepatic mRNA level. Thirdly, RXRα binding can be “silent”. Active gene transcription occurs upon ligand binding, which leads to recruitment of many other factors and Pol II. It is possible that the biological level of RXRα ligands is not sufficient to induce gene transactivation and thus knockout RXRα has no impact on basal mRNA level. Additional experiments that use pharmacological ligands such as retinoic acids and polyunsaturated fatty acids to activate RXRα are needed to identify exogenous ligand/RXRα-dependent genes. The expression of gene is tissue specific. Figure 1A shows that there are 7642 genes have RXRα binding, but mRNA levels of those genes cannot be detected in the liver. Those genes might be expressed in the non-hepatic tissues such as neuron and skeletal muscle. We speculate that RXRα might have the most extensive binding in the liver genome since it has the highest expression level in the liver. Those binding sites may also exist in the DNA derived from other tissues.

As a master nuclear receptor, RXRα plays an essential role in effecting the biological actions of other adopted orphan nuclear receptors. Based on the “1–2–3–4–5 rule”, RXR and its partners preferentially bind to specific motifs composed of hexamers (A/GGGTCA) separated by a various number of nucleotides [45]. For example, DR1 is a preferred motif for peroxisome proliferator activator receptors (PPAR)/RXR heterodimer and RXR homodimer. DR4 is the preferred binding site for liver x receptor and thyroid hormone receptor [1], [46], and IR1 is the preferred motif for farnesoid x receptor [47]. DR3, DR4, and ER6 are usually recognized by pregnane x receptor [48]. DR2 and 5 are the main binding motifs for retinoic acid receptor [1], [45]. Therefore, the specific heterodimeric partners of RXRα that are involved in the regulatory process can be predicted based on the motif. The present data showed that DR1 was the most common motif, followed by DR4, IR1, and DR3. According to the “1–2–3–4–5 rule” mentioned above, PPAR/RXRα heterodimer and RXRα homodimer may be the dominant regulators followed by LXR, FXR, and PXR in the liver. In agreement with this finding, a recent study showed that 67.7% and 25.7% of RXRα binding sites overlap with those of PPARα and LXR, respectively, in normal mouse livers [49]. Furthermore, the finding is consistent with the phenotype of hepatocyte RXRα-deficient mice, which have elevated serum cholesterol and triglyceride levels [16]. In addition, lipid, bile acid, and xenobiotic metabolism are altered when the hepatocyte RXRα-deficient mice are challenged with exogenous ligands for those receptors [10], [12]. Notably, analysis of common sequences of the 500 strongest RXRα peaks demonstrated that GC box, the motif of Sp1, may be one of the most popular motifs for RXRα binding. Consistently, it has been shown that there is a thyroid hormone response element overlapping with the GC box in the promoter of the epidermal growth factor receptor, where it is bound competitively by RXR/T3R and Sp1 [42]. Furthermore, a physical interaction between RAR/RXR and Sp1 has also been reported and such interaction synergistically enhances the expression of RA-induced genes *in vitro* [50]. Our findings suggest the presence of extensive crosstalk between Sp1 and RXRα and their collaborative or competitive regulatory role in regulating liver gene transcription.

Recent studies have reported the mouse liver genome-wide binding profile of FXR [26], PXR [33], LXR, and PPARα [49]. Comparing the RXRα ChIP-seq data from this study with others' findings, common bindings were aligned between RXRα and its partners (data not shown). Accordingly, biological functional analysis of RXRα-bound genes (Table 3) encompassed almost all its partners' biological functions. In addition, we identified novel pathways that are specific for RXRα, which include oxidation/reduction, protein localization, intracellular signaling cascade, regulation of transcription, cofactor metabolic process, cellular response to stress, and cell death. Furthermore, novel potential of RXRα was also unveiled by the analysis of RXRα-bound non-hepatic genes. Based on binding and expression profiling generated from wild type and knockout mice, this paper is the first to establish the relationship between binding and expression in an RXRα-dependent manner.

It is important to note that our results did not show any significant correlation between peak score and the fold change in mRNA level caused by RXRα deficiency. In addition, the binding characteristic (location of the peak) is similar between UIK and DIK genes. This finding suggests that physical interaction of RXRα is essential, but not sufficient for predicting the subsequent transcriptional effect.

The role of retinoids in other organs such as the eye and skin is well known. However, retinoids are stored, processed, and metabolized in the liver. In addition, the liver is also a retinoid target organ and yet the action of retinoids in the liver has been overlooked. This study, for the first time, demonstrates the potential biological effect of endogenous ligands of RXRα in the liver. Taken together, as an active partner of many nuclear receptors, our reported data showed that RXRα and its endogenous ligands control liver metabolism and function in general.
